# PIM-1 mRNA expression is a potential prognostic biomarker in acute myeloid leukemia

**DOI:** 10.1186/s12967-017-1287-4

**Published:** 2017-08-29

**Authors:** Hui Cheng, Chongmei Huang, Xiaoqian Xu, Xiaoxia Hu, Shenglan Gong, Gusheng Tang, Xianmin Song, Weiping Zhang, Jianmin Wang, Li Chen, Jianmin Yang

**Affiliations:** 1 Institute of Hematology, Changhai Hospital, Second Military Medical University, Shanghai, 200433 China; 2 Department of Hematology, Shanghai First People’s Hospital, Shanghai Jiaotong University, Shanghai, 200433 China

**Keywords:** PIM-1, mRNA, Expression, Prognosis, Acute myeloid leukemia

## Abstract

**Background:**

High expression of proviral integration site for Moloney murine leukemia virus-1 (PIM-1), a serine/threonine kinase, is associated with many cancers. The main purpose of this study were to investigate that the correlation between PIM-1 mRNA levels and clinicopathologic features and its clinical significance in acute myeloid leukemia (AML).

**Methods:**

qRT-PCR was performed for 118 de novo AML and 20 AML complete remission patients and 15 normal individuals. All statistical analysis were performed using Graphpad Prism5 software.

**Results:**

We observed that expression of PIM-1 mRNA was higher in AML patients than in healthy individuals and in complete remission AML patients (*P* = 0.0177). Further, high PIM-1 mRNA levels were more associated with high-risk FLT3+ AML patients than the FLT3− group (*P* = 0.0001) and were also associated with clinical factors such as risk stratification (*P* = 0.0029) and vital status (*P* = 0.0322). Kaplan–Meier survival analysis indicated that PIM-1 mRNA expression correlated with overall survival (OS), disease free survival (DFS), and relapse rate (RR) in AML patients. Most importantly, the high PIM-1-expressing patients took longer to achieve complete remission than the low expression group (*P* = 0.001). In addition, the complete remission rate was significantly lower in the high PIM-1 group (*P* = 0.0277) after induction therapy.

**Conclusions:**

Above results suggest that PIM-1 mRNA levels may be an independent prognostic factor in AML.

**Electronic supplementary material:**

The online version of this article (doi:10.1186/s12967-017-1287-4) contains supplementary material, which is available to authorized users.

## Background

The PIM-1 gene is a proto-oncogene located on chromosome 6p21 that encodes a serine/threonine kinase, found mostly in hematopoietic cells as a member of the PIM family [1]. It was originally identified as a proviral integration site for Moloney murine leukemia virus-1 [2]. PIM belongs to a family with three closely related members (PIM-1, PIM-2, and PIM-3), which are constitutively active serine/threonine kinases that promote cell survival, proliferation and migration in several malignancies including cancers [3–5]. PIM-1 tightly regulates its own expression and function [6, 7], and also regulates critical signaling pathways like JAK/STAT [3] and PI3 K/AKT [8]. The PIM-1 gene encodes two isoforms, PIM-1L and PIM-1S, as a result of translation initiation at two different sites [2]. PIM-1L is the longer isoform with a molecular mass of 44 kDa and mainly localizes to plasma membrane; PIM-1S is the shorter isoform with a molecular mass of 33 kDa, which predominately localizes into nucleus and cytosol. These 2 isoforms have distinct functions in various malignancies [9]. Moreover, PIM-1 is also upregulated in advanced prostate cancer and esophageal squamous cell carcinoma [10, 11].

Acute myeloid leukemia (AML) is a biologically complex and clinically heterogeneous disease that accounts for nearly 80% of adult acute leukemia cases with increased incidence with age [12]. A number of cytogenetic and molecular aberrations have been identified and form the basis of classification and risk stratification of AML [13]. In 97% of AML cases, genetic mutations have been identified, often presented as cytogenetically normal (CN-AML) AML [14, 15]. Cytogenetics and mutation testing are the most important prognostic tools for AML treatment after induction therapy [16]. Although there is rapid improvement in risk stratification of AML gene mutations and development of many new drugs such as monoclonal anti-CD33 antibody, inhibitor of mutant IDH1 and IDH2 and inhibitor of DOT1L, majority of patients still experience relapse and succumb to the disease [17–20]. The 5-year survival rate stands at 26% after AML diagnosis, and the extremely poor survival rate indicates the aggressive nature of this malignant disease [21]. Recently, studies focused on the mechanism of PIM-1 transcription and AML [22, 23]. Until now, little data is known regarding the impact of PIM-1 mRNA overexpression on prognosis in AML patients.

In this retrospective study, we examined the expression of PIM-1 at the mRNA level and explored the relationship of PIM-1 expression with clinicopathologic parameters, including overall survival. We found that the expression of PIM-1 was associated with poor prognosis of AML patients.

## Methods

### Patients and clinical samples

The study was approved by the Medical Ethical Committee of First Affiliated Hospital of Second Military Medical University, China. All 118 patients received induction therapy with either typically “7 + 3” or a similar regimen with daunorubicin and cytarabine-based induction and consolidation therapies. The “7 + 3” regiment consisted of the following agents: idarubicin (8–12 mg/m^2^/day × 3 days) or daunorubicin (45–90 mg/m^2^/day × 3 days), cytarabine (100–200 mg/m^2^/day × 7 days). Leukemia cell samples were obtained from 118 new diagnosis patients between January 2010 and December 2014 from the Department of Hematology, First Affiliated Hospital of Second Military Medical University, China. All patients gave their informed consent for this study. The patient samples were selected in this study only if follow up was obtained and all clinical data were available. Meanwhile, bone marrow samples from complete remission AML patients (n = 20) and normal individuals without any blood diseases (n = 15) were also obtained for this study. Mononuclear cell isolation was performed using Lymphoprep (Hao-Yang Biological Manufacture Co., Ltd., TianJin, China).

### RNA extraction and qRT-PCR

Total RNA was extracted from bone marrow mononuclear cells isolated from AML and complete remission AML patients and normal individuals as controls using Trizol (Invitrogen; Carlsbad, CA, USA) according to the manufacturer’s instructions. The RNA was quantified and dissolved with DEPC water. 1ìg RNA was converted to cDNA using a cDNA synthesis kit (Takara Bio, Inc., Tokyo, Japan). The content of kit includes PrimeScript RTase, RNase Inhibitor, Random 6 mers, Oligo dT Primer, dNTP Mixture and Buffer, and reaction conditions were as follows: incubation for 15 min at 37 °C, inactivation 5 s at 85 °C. qRT-PCR was performed in the ABI 7500 amplification system (Applied Biosystems, Foster City, CA, USA) using the cDNA samples. Glyceraldehyde 3-phosphate dehydrogenase (GAPDH) was used as an internal control. The quantitative PCR primers were as follows: PIM-1 forward, 5′-GCAAATAGCAGCCTTTCTGG-3′ and reverse 5′-CCTAGGACCCCTGGAGAGTC-3′; GAPDH forward, 5′-CTCCTGTTCGACAGTCAGCC-3′ and reverse 5′-TTCCCGTTCTCAGCCTTGAC-3′. The SYBR Premix Ex Taq II kit (Takara Bio, Inc., Tokyo, Japan) was used for real-time qPCR. The PCR conditions were as follows: 40 cycles of pre-incubation for 30 s at 95 °C, denaturation for 5 s at 95 °C and annealing for 34 s at 60 °C. The amplification plots were shown in Additional file 1: Figure S1. The 2^−△Ct^ method was used to quantify the relative PIM-1 mRNA levels normalized to GAPDH mRNA. Calculated results were shown by 2^−△Ct^ (%).

### Patient classification and risk stratification

AML patients were classified as favorable, intermediate or adverse groups according to 2012 European LeukemiaNet clinical practice guidelines based on chromosomal fusion and gene mutations (Table 1) [13].

**Table 1:** 2012 European LeukemiaNet prognostic-risk group based on cytogenetic and molecular profile

Prognostic-risk group	Cytogenetic profile alone	Cytogenetic profile and molecular abnormalities
Favorable	t(8:21)(q22; q22) inv(16)(p13; 1q22) t(15;17)(q22; q12)	t(8:21)(q22; q22) with no c-KIT mutation inv(16)(p13; 1q22) t(15;17)(q22; q12) Mutated NPM1 without FLT3-ITD (CN-AML) Mutated biallelic CEBPA (CN-AML)
Intermediate	CN-AML t(9;11)(p22; q23) Cytogenetic abnormalities not included in the favorable or adverse prognostic risk groups	t(8:21)(q22;q22) with mutated c-KIT CN-AML other than those included in the favorable or adverse prognostic group t(9;11)(p22; q23) Cytogenetic abnormalities not included in the favorable or adverse prognostic risk groups
Adverse	inv(3)(q21q26.2) t(6;9)(p23; q34) 11q abnormalities other than t(9;11) −5 or del(5q) −7 Complex karyotype	TP53 mutation, regardless of cytogenetic profile CN with FLT3-ITD CN with DNMT3A CN with KMT2A-PTD inv(3)(q21q26.2) t(6;9)(p23;q34) 11q abnormalities other than t(9;11) −5 or del(5q) −7 Complex karyotype

*CN-AML* normal cytogenetics acute myeloid leukemia, *ITD* internal tandem duplications

### Follow-up

Clinical and laboratory examinations were used for the follow-up of AML patients, including cell morphology, immunology, cytogenetics and molecular biology. Overall survival was defined as the time from the date of treatment to the date of death or the last follow-up examination. In the current study, out of the 118 patients, 88 patients died during the follow-up period.

### Statistical analysis

Event-free survival (EFS) was defined as the time from the date of diagnosis to removal from the study due to relapse, disease progression or death. Disease-free survival (DFS) refers to the time from randomization to relapse or death due to disease progression. Overall survival (OS) is the time from randomization to death for any cause. Time to complete remission refers to the time from the date of diagnosis to acquisition morphological less than 5% of the leukemia burden by chemotherapy.

All statistical analysis was performed using Graphpad Prism5 software. The Student’s t test and one way ANOVA were used to analyze the correlation between PIM-1 mRNA expression and the clinicopathological features in AML patients. Complete remission rate was analyzed by Chi square test. Kaplan–Meier plots were generated for patient survival and analyzed using the Mantel–Cox log-rank test. *P* value <0.05 was considered statistically significant.

## Results

### Clinical characteristics of patients with AML

In present study, we evaluated 118 de novo AML patients, 35 AML complete remission patients and normal individuals as controls. The 118 patients with AML included 62 males and 56 females with a median age of 47 years (range 14–72 years). We used the 2012 European LeukemiaNet clinical practice guidelines to classify the patients into 49 favorable, 28 intermediate and 41 adverse cases. Allogenic hematopoietic stem cell transplantation had been performed on 31 patients and 87 patients had received chemotherapy alone. The median follow-up time of surviving patients was 12 months (range 0.2–77 months). The clinicopathologic characteristics of the patients are listed in Table 2.

**Table 2:** Correlation between PIM-1 expression and clinicopathological features in patients with acute myeloid leukemia (AML)

Clinicopathological feature	Total	mRNA expression of PIM-1		*P* value
		Low (n = 38, 32.2%)	High (n = 80, 67.8%)	
Gender				
Male	62	17 (27.5%)	45 (72.5%)	0.1522
Female	56	21 (37.5%)	35 (62.5%)	
Age (years)				
≤47	67	21 (31.3%)	46 (68.7%)	0.3469
>47	51	17 (33.3%)	34 (66.7%)	
WBC (×10^9^)				
<10	57	16 (28.1%)	41 (71.9%)	0.9206
>10	61	22 (36.1%)	39 (63.9%)	
Hb (g/L)				
<81	66	18 (27.3%)	48 (72.7%)	0.7689
>81	52	20 (38.5%)	32 (61.5%)	
PLT (×10^9^)				
<57	75	28 (37.3%)	47 (62.7%)	0.7321
>57	43	10 (23.3%)	33 (76.7%)	
Blasts (%)				
<62	54	14 (25.9%)	40 (74.1%)	0.1762
>62	64	24 (37.5%)	40 (62.5%)	
FAB classification				
M1	4	1 (25%)	3 (75%)	0.1162
M2	35	15 (42.9%)	20 (57.1%)	
M4	37	11 (29.7%)	26 (70.3%)	
M5	37	9 (24.3%)	28 (75.7%)	
M6	5	2 (40%)	3 (60%)	
Risk stratification				
Low risk	49	24 (48.9%)	25 (51.1%)	0.0029^a^
Intermediate risk	28	9 (32.1%)	19 (67.2%)	
High risk	41	5 (12.2%)	36 (87.8%)	
Treatment				
Chemotherapy	87	26 (29.9%)	61 (70.1%)	0.9098
HSCT	31	12 (38.7%)	19 (61.3%)	
Vital status				
Survival	27	14 (51.9%)	13 (48.1%)	0.0322^a^
Death	91	24 (26.4%)	67 (73.6%)	

*HSCT* hematopoietic stem cell transplant

^a^Statistically significant

### Relationship between PIM-1 mRNA and clinical features in AML patients

**Fig. 1:**
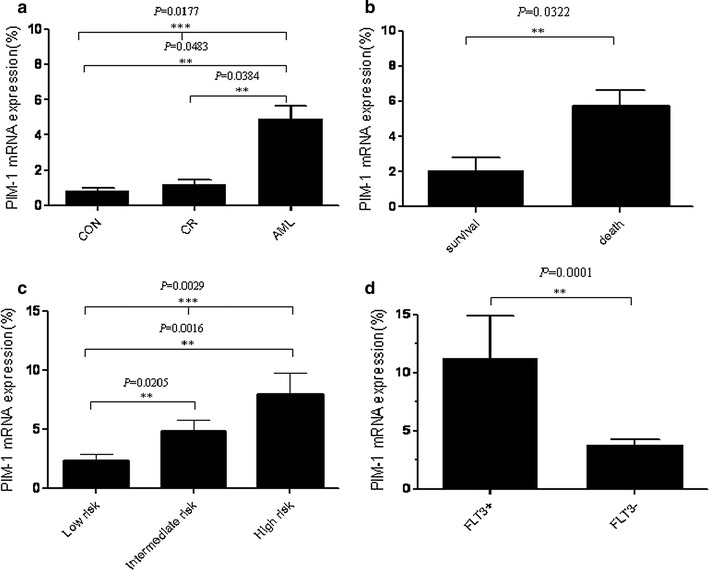
PIM-1 mRNA expression is increased in AML. The one-way ANOVA analysis shows the significant differences of PIM-1 mRNA expression in AML patients compared with complete remission samples and normal control samples (**a**) and the significant differences among low risk, intermediate risk and high risk (**c**). The student’s t test results show PIM-1 mRNA expression is obviously upregulated in FLT3+ AML patients and died patients compared with FLT3− patients and surviving patients (**b**, **d**), respectively

We first analyzed PIM-1 mRNA expression in samples from 118 de novo AML and 20 AML complete remission patients and 15 normal individuals by qRT-PCR. The results indicated that PIM-1 mRNA was significantly increased 4–5 folds in 118 AML patients compared to AML complete remission and normal control individuals, respectively (*P* = 0.0177, Fig. 1a).

We further analyzed the association between PIM-1 mRNA levels and various clinicopathologic features including gender, age, white blood cell counts, hemoglobin content, platelet counts,  % blasts, FLT3 status, FAB classification risk stratification, treatment and vital status by Student’s t test and one-way ANOVA. As shown in Fig. 1b–d, PIM-1 mRNA levels were significantly correlated with risk stratification (*P* = 0.0029), FLT3 (− or +; *P* = 0.0001) and vital status (*P* = 0.0322). As shown in Table 2, no significant correlation was found between PIM-1 mRNA levels and clinical features such as gender, age, white blood cells, hemoglobin, platelet, blasts (%), FAB classification and treatment.

### High PIM-1 mRNA levels predicts poor prognosis for AML patients

**Fig. 2:**
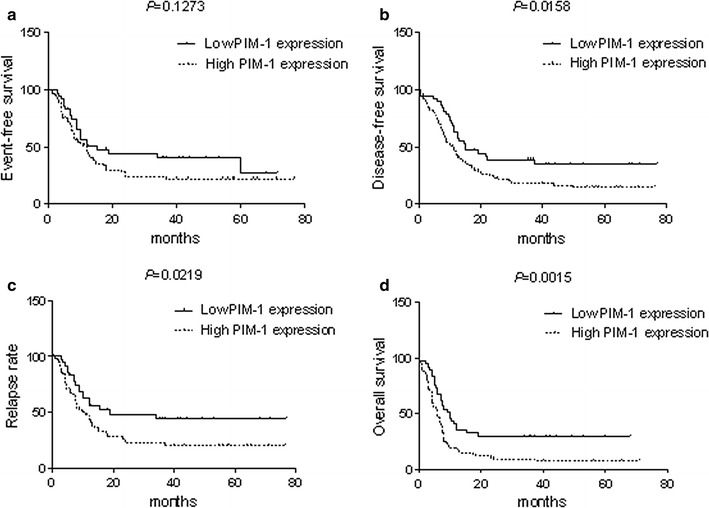
The correlation between PIM-1 mRNA expression and various prognostic parameter. Kaplan–Meier survival analysis for the difference between curves of PIM-1 low-expression and high-expression patients was compared in event-free survival (**a**), disease-free survival (**b**), relapse rate (**c**) and overall survival (**d**). *P* value is calculated according to log-rank test

**Fig. 3:**
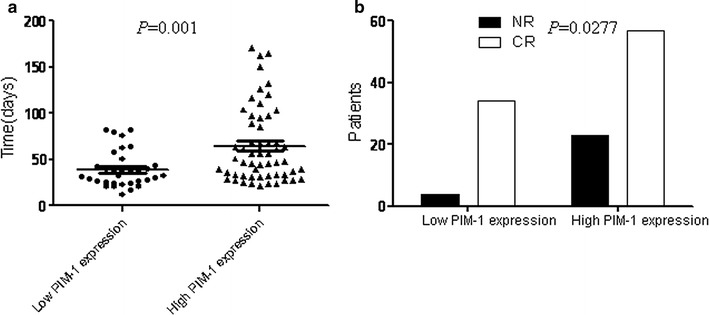
The comparisons of time (days) of obtaining complete remission and complete remission rate between low and high PIM-1 expression. Comparisons of the time (days) of obtaining complete remission between low and high expression in patients with AML (**a**). Comparisons of complete remission rate between low and high expression in patients with AML after induction therapy (**b**). P value is calculated according to student’s t test (**a**). *P* value is calculated according to Chi square test (**b**)

**Fig. 4:**
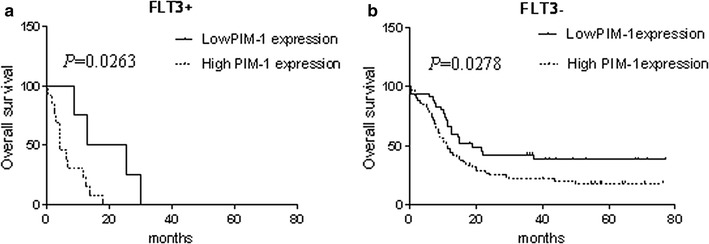
The difference of over survival between low and high PIM-1 expression in FLT3 (∓) patients. Kaplan–Meier survival curves show the statistical differences in over survival between low and high PIM-1 mRNA expression in FLT3+ (**a**) or FLT3− (**b**) AML patients. *P* value is calculated according to log-rank test

**Fig. 5:**
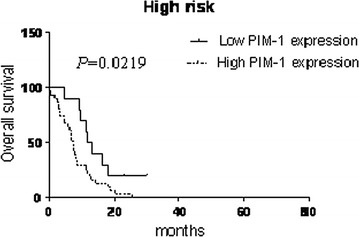
The comparison of over survival between low and high PIM-1 expression in high risk patients with AML. Kaplan–meier survival analysis shows the significant difference between low and high expression in high risk patients with AML. *P* value is calculated according to log-rank test

We further evaluated if PIM-1 mRNA was a prognostic indicator in AML patients by analyzing PIM-1 expression levels with the clinical follow-up information based on Kaplan–Meier analysis and log-rank test. Based on ROC curve, PIM-1 expression level at the value of 0.5424 (%) was selected as the cut-off value to define lower- and higher-expression groups. As shown in Fig. 2, AML patients with higher PIM-1 expression had significant correlation with DFS (*P* = 0.0158), RR (*P* = 0.0219) and OS (*P* = 0.0015) compared to those with lower PIM-1 expression and no significant difference in EFS (Additional file 2: Table S1). Further, we observed that the high PIM-1 expression group needed significantly longer time for complete remission than the low PIM-1 expression group (*P* = 0.001, Fig. 3a). Also, the complete remission rate was significantly lower in high PIM-1 group (71.25%) than the low expression group (89.47%) after induction therapy (*P* = 0.0277; Fig. 3b). The OS of high PIM-1 expression group was significantly lower compared to that of the low PIM-1 expression group in the FLT3+ and FLT3− categories (Fig. 4). The OS of low PIM-1 expression group is better than that of high expression group in the high risk AML patients (Fig. 5). These results indicated that high PIM-1 expression suggested poor prognosis in AML.

## Discussion

AML is a heterogeneous disease that is characterized by clonal expansion of undifferentiated myeloid precursors, which ultimately results in impaired hematopoiesis and bone marrow failure [24]. Although 60–80% of patients with de novo AML achieve complete remission with induction therapy, significant number of cases undergo relapse that accounts for the poor overall long-term survival rate [16, 25]. Hence, accurate assessment of prognosis is central to the physician deciding between standard or elevated treatment intensity; consolidation chemotherapy or allogenic hematopoietic stem cell transplant; established or investigational therapy [26, 27]. Therefore we investigated if PIM-1 mRNA expression levels can be prognostic in AML by evaluating its association with various clinical and prognosis parameters in AML patients.

Previous studies have indicated that PIM-1 kinase has a critical role in tumorigenesis [1, 10, 28]. It is an important anti-tumor therapeutic target because it interacts with various proteins and regulates many critical signaling pathways [9]. Oncogenic FLT3-ITD was shown to aberrantly activate STAT5 that promoted expression of PIM-1 kinase [29]. The PIM-1 kinase is an integral part of FLT3 signaling pathway and phosphorylates many pro-apoptotic proteins; in some cases it may act upstream of FLT3 [22, 30]. Recent studies have demonstrated that PIM-1 transcription is regulated by IL-6 and FOXP3, which suggests that PIM-1 can be a potential target for immunotherapy [31, 32]. Also, there is evidence that PIM-1 mediates drug resistance through its interaction with FLT3 [23, 33]. This suggests that PIM-1 is part of a complex network that regulates tumor progression in AML and elevated PIM-1 expression maybe of prognostic significance to AML patients. Besides, Goldberg et al. studied that PIM-1 and the RAS pathway are potential therapeutic targets of high-risk leukemia [34]. In addition, ETS2, a downstream effector of the RAS/RAF/ERK pathway, the same as PIM-1, which plays a crucial role in cell growth and Fu et al. also reported that high expression of ETS2 predicted poor prognosis in acute myeloid leukemia [35, 36]. This illustrates that our finding may be relevant to these studies.

Our data was consistent with previous findings [23], which shown that PIM-1 was highly expressed in AML patients with FLT3+ compared with FLT3− patients (76.47% vs 45.54%, *P* = 0.0183; Additional file 1: Figure S1). Furthermore, PIM-1 mRNA was significantly higher in AML patients compared to both the complete remission patients and normal control group (Fig. 1a). The current study demonstrated that PIM-1 high expression indicates poor prognosis in FLT3+ and FLT3− AML patients (Fig. 4) and PIM-1 expression was higher in FLT3+ AML patients than FLT3− (Fig. 1d). Also, it significantly correlated with clinical factors like efficacy and risk stratification suggesting its potential as an independent biomarker. Kaplan–Meier analysis revealed that the AML patients with low PIM-1 expression had significantly prolonged overall survival compared with high PIM-1 expressing patients. Consistent with the findings in solid tumor, PIM-1 mRNA expression is also up-regulated during malignant transformation in prostate cancer and esophageal squamous cell carcinoma [11, 37].

## Conclusions

Our data indicated that PIM-1 mRNA expression levels are significantly correlated with prognosis, and might be an independent prognostic factor in AML. Simultaneously, the results also might provide a novel biomarker that can be used to diagnose and monitor minimal residual disease in AML.

## Additional files

**Additional file 1.** Additional figure.

**Additional file 2.** The comparisons of the number of patients with PIM-1 high expression between FLT3+ and FLT3− group. *P* value is calculated according to student’s t test.

## Abbreviations

PIM-1: proviral integration site for Moloney murine leukemia virus-1
AML: acute myeloid leukemia
qRT-PCR: real time quantitative polymerase chain reaction
FLT3: fms-like tyrosine kinase
CR: complete remission
OS: overall survival
DFS: disease free survival
RR: relapse rate
CN-AML: cytogenetically normal acute myeloid leukemia
ROC: receiver operating characteristic
EFS: event-free survival

## References

[1] Narlik-Grassow M, Blanco-Aparici C, Carnero A (2014). The PIM family of serine/threonine kinases in cancer. Med Res Rev.

[2] Saris CJ, Domen J, Berns A (1991). The pim-1 oncogene encodes two related protein-serine/threonine kinases by alternative initiation at AUG and CUG. EMBO J.

[3] Magnuson NS, Wang Z, Ding G, Reeves R (2010). Why target PIM-1 for cancer diagnosis and treatment?. Future Oncol.

[4] Santio NM, Vahakoski RL, Rainio EM, Sandholm JA, Virtanen SS, Prudhomme M (2010). Pim selective inhibitor DHPCC-9 reveals Pim kinases as potent stimulators of cancer cell migration and invasion. Mol Cancer.

[5] Santio NM, Eerola SK, Paatero I, Yli-Kauhaluoma J, Anizon F, Moreau P (2015). Pim kinases promote migration and metastatic growth of prostate cancer xenografts. PLoS ONE.

[6] Zhao Y, Hamza MS, Leong HS, Lim CB, Pan YF, Cheung E (2008). Kruppel-like factor 5 modulates p53 independent apoptosis through Pim-1 survival kinase in cancer cells. Oncogene.

[7] Santio NM, Salmela M, Arola H, Eerola SK, Heino J, Rainio EM (2016). The PIM-1 kinase promotes prostate cancer cell migration and adhesion via multiple signaling pathways. Exp Cell Res.

[8] Cen B, Mahajan S, Wang W, Kraft AS (2013). Elevation of receptor tyrosine kinases by small molecule AKT inhibitors in prostate cancer is mediated by Pim-1. Cancer Res.

[9] Tursynbay Y, Zhang J, Li Z, Tokay T, Zhumadilov Z, Wu D (2016). Pim-1 kinase as cancer drug target: an update. Biomed Rep.

[10] Warfel NA, Kraft AS (2015). PIM kinase (and Akt) biology and signaling in tumors. Pharmacol Ther.

[11] Liu HT, Wang N, Wang X, Li SL (2010). Overexpression of Pim-1 is associated with poor prognosis in patients with esophageal squamous cell carcinoma. J Surg Oncol.

[12] Yamamoto JF, Goodman MT (2008). Patterns of leukemia incidence in the United States by subtype and demographic characteristics, 1997–2002. Cancer Causes Control.

[13] Mrózek K, Marcucci G, Nicolet D, Maharry KS, Becker H, Whitman SP (2012). Prognostic significance of the European Leukemia Net standardized system for reporting cytogenetic and molecular alterations in adults with acute myeloid leukemia. J Clin Oncol.

[14] Patel JP, Gönen M, Figueroa ME, Fernandez H, Sun Z, Racevskis J (2012). Prognostic relevance of integrated genetic profiling in acute myeloid leukemia. N Engl J Med.

[15] Cancer Genome Atlas Research Network (2013). Genomic and epigenomic landscapes of adult de novo acute myeloid leukemia. N Engl J Med.

[16] Döhner H, Weisdorf DJ, Bloomfield CD (2015). Acute myeloid leukemia. N Engl J Med.

[17] Medinger M, Lengerke C, Passweg J (2016). Novel prognostic and therapeutic mutations in acute myeloid leukemia. Cancer Genom Proteom.

[18] Abutalib SA, Tallman MS (2006). Monoclonal antibodies for the treatment of acute myeloid leukemia. Curr Pharm Biotechnol.

[19] Stein EM (2015). IDH2 inhibition in AML: finally progress?. Best Pract Res Clin Haematol.

[20] Rau RE, Rodriguez BA, Luo M, Jeong M, Rosen A, Rogers JH (2016). DOT1L as a therapeutic target for the treatment of DNMT3A mutant acute myeloid leukemia. Blood.

[21] American Cancer Society. Cancer facts and figures 2016. https://old.cancer.org/acs/groups/content/@research/documents/document/acspc-047079.pdf.

[22] Kim KT, Levis M, Small D (2006). Constitutively activated FLT3 phosphorylates BAD partially through pim-1. Br J Haematol.

[23] Kim KT, Baird K, Ahn JY, Meltzer P, Lilly M, Levis M (2005). Pim-1 is up-regulated by constitutively activated FLT3 and plays a role in FLT3− mediated cell survival. Blood.

[24] Papaemmanuil E, Gerstung M, Bullinger L, Gaidzik VI, Paschka P, Roberts ND (2016). Genomic clssification and prognosis in acute myeloid leukemia. N Engl J Med.

[25] Büchner T, Schlenk RF, Schaich M, Döhner K, Krahl R, Krauter J (2012). Acute myeloid leukemia (AML): different treatment strategies versus a common standard arm-combined prospective analysis by the German AML Intergroup. J Clin Oncol.

[26] De Kouchkovsky I, Abdul-Hay M (2016). Acute myeloid leukemia: a comprehensive review and 2016 update. Blood Cancer J.

[27] Schetelig J, Schaich M, Schäfer-Eckart K, Hänel M, Aulitzky WE, Einsele H (2015). Study-Alliance-Leukemia: hematopoietic cell transplantation in patients with intermediate and high risk AML: results from the randomized Study Alliance Leukemia (SAL) AML 2003 trial. Leukemia.

[28] Chen J, Kobayashi M, Darmanin S, Qiao Y, Gully C, Zhao R (2009). Hypoxia-mediated up-regulation of Pim-1 contributes to solid tumor formation. Am J Pathol.

[29] Mizuki M, Schwable J, Steur C, Choudhary C, Agrawal S, Sargin B (2003). Suppression of myeloid transcription factors and induction of STAT response genes by AML specific Flt3 mutations. Blood.

[30] Fathi AT, Arowojolu O, Swinnen I, Sato T, Rajkhowa T, Small D (2012). A potential therapeutic target for FLT3-ITD AML: PIM-1 kinase. Leuk Res.

[31] Block KM, Hanke NT, Maine EA, Baker AF (2012). IL-6 stimulates STAT3 and Pim-1 kinase in pancreatic cancer cell lines. Pancreas.

[32] Li Z, Lin F, Zhuo C, Deng G, Chen Z, Yin S, Gao Z (2014). PIM-1 kinase phosphorylates the human transcri-ption factor FOXP3 at serine 422 to negatively regulate its activity under inflammation. J Biol Chem.

[33] Natarajan K, Bhullar J, Shukla S, Burcu M, Chen ZS, Ambudkar SV (2013). The Pim kinase inhibitor SGI-1776 decreases cell surface expression of P-glycoprotein (ABCB1) and breast cancer resistance protein (ABCG2) and drug transport by Pim-1 dependent and independent mechanisms. Biochem Pharmacol.

[34] Goldberg L, Tijssen MR, Birger Y, Hannah RL, Kinston SJ, Schütte J (2013). Genome-scale expression and transcription factor binding profiles reveal therapeutic targets in transgenic ERG myeloid leukemia. Blood.

[35] Bonin S, Larese FF, Trevisan G, Avian A, Rui F, Stanta G (2011). Gene expression changes in peripheral blood mononuclear cells in occupational exposure to nickel. Exp Dermatol.

[36] Fu L, Fu H, Wu Q, Pang Y, Xu K, Zhou L (2017). High expression of ETS2 predicts poor prognosis in acute myeloid leukemia and may guide treatment decisions. J Transl Med.

[37] Wu YB, Lu D, He ZF, Jin CG (2016). PIM-1 polymorphism and PIM-1 expression as predisposing factors of esophageal squamous cell carcinoma in the Asian population. Onco Targets Ther.

