# Supplementary figures and images for: PIM-1 mRNA expression is a potential prognostic biomarker in acute myeloid leukemia
Source: J Transl Med. 2017 Aug 29;15:179. doi: 10.1186/s12967-017-1287-4 (PMC5576275; doi:10.1186/s12967-017-1287-4)

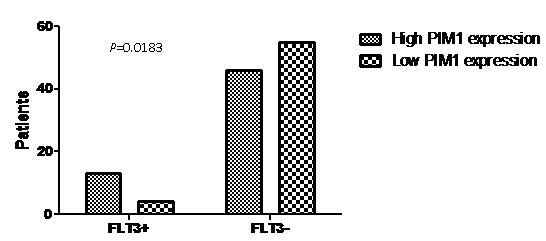

Supplement: Supplementary file 1 — Additional file 1. Additional figure. [file 12967_2017_1287_MOESM1_ESM.tif]
