# Supplementary material for: PIM-1 mRNA expression is a potential prognostic biomarker in acute myeloid leukemia
Source: J Transl Med. 2017 Aug 29;15:179. doi: 10.1186/s12967-017-1287-4 (PMC5576275; doi:10.1186/s12967-017-1287-4)
Supplement: Supplementary file 2 — Additional file 2. The comparisons of the number of patients with PIM-1 high expression between FLT3+ and FLT3− group. P value is calculated according to student’s t test. [file 12967_2017_1287_MOESM2_ESM.doc]

| Prognostic parameter | *P* -value | 95%CI |
| --- | --- | --- |
| Event free survival (EFS) | 0.1273 | 0.6938-1.889 |
| Disease free survival (DFS) | **0.0158*** | 0.7060-1.961 |
| Over survival (OS) | **0.0015*** | 1.029-2.3050 |
| Relapse rate (RR) | **0.0219*** | 1.316-2.4840 |
| Treatment relative mortality (TRM) | 0.6777 | 0.0619-0.9726 |

**Additional file 2: Table S1** The analysis of prognosis in AML patients between high expression of PIM-1 mRNA group and low expression group.

*****statistically significant.
